# PFKFB3 Connects Glycolytic Metabolism with Endothelial Dysfunction in Human and Rodent Obesity

**DOI:** 10.3390/antiox14020172

**Published:** 2025-01-31

**Authors:** Robert K. Batori, Zsuzsanna Bordan, Caleb A. Padgett, Yuqing Huo, Feng Chen, Reem T. Atawia, Rudolf Lucas, Masuko Ushio-Fukai, Tohru Fukai, Eric J. Belin de Chantemele, David W. Stepp, David J. R. Fulton

**Affiliations:** 1 Vascular Biology Center, Medical College of Georgia, Augusta University, Augusta, GA 30912, USA; rbatori@augusta.edu (R.K.B.); zbordan@augusta.edu (Z.B.); capadgett@augusta.edu (C.A.P.); rlucas@augusta.edu (R.L.); mfukai@augusta.edu (M.U.-F.); tfukai@augusta.edu (T.F.); ebelindechanteme@augusta.edu (E.J.B.d.C.); dstepp@augusta.edu (D.W.S.); 2 Department of Ophthalmology, Baylor College of Medicine, Houston, TX 77030, USA; yuqing.huo@bcm.edu; 3 Department of Forensic Medicine, Nanjing Medical University, Nanjing 210029, China; fchen@njmu.edu.cn; 4 Department of Pharmaceutical Sciences, College of Pharmacy, Southwestern Oklahoma State University, Weatherford, OK 73096, USA; reem.atawia@swosu.edu; 5 Department of Pharmacology and Toxicology, Faculty of Pharmacy, Ain Shams University, Cairo 11566, Egypt; 6 Department of Pharmacology and Toxicology, Medical College of Georgia, Augusta University, Augusta, GA 30912, USA; 7 Department of Medicine (Cardiology), Medical College of Georgia, Augusta University, Augusta, GA 30912, USA; 8 Charlie Norwood Veterans Affairs Medical Center, Augusta, GA 30912, USA; 9 Department of Physiology, Medical College of Georgia, Augusta University, Augusta, GA 30912, USA

**Keywords:** PFKFB3, eNOS, nitric oxide, ROS, NOX1, NOX5, type 2 diabetes

## Abstract

Obesity and type 2 diabetes (T2D) increase cardiovascular risk, largely due to altered metabolic state. An early consequence of T2D/obesity is the loss of endothelial function and impaired nitric oxide (NO) signaling. In blood vessels, endothelial nitric oxide synthase (eNOS) synthesizes NO to maintain vessel homeostasis. The biological actions of NO are compromised by superoxide that is generated by NADPH oxidases (NOXs). Herein we investigated how altered metabolism affects superoxide/NO balance in obesity. We found that eNOS expression and NO bioavailability are significantly decreased in endothelial cells (ECs) from T2D patients and animal models of obesity. In parallel, PFKFB3, a key glycolytic regulatory enzyme, is significantly increased in ECs of obese animals. EC overexpression of wild-type and a cytosol-restricted mutant PFKFB3 decreased NO production due to increased eNOS-T495 phosphorylation. PFKFB3 also blunted Akt-S473 phosphorylation, reducing stimulus-dependent phosphorylation of S1177 and the activation of eNOS. Furthermore, PFKFB3 enhanced the activities of NOX1 and NOX5, which are major contributors to endothelial dysfunction. Prolonged exposure of ECs to high glucose or TNFα, which are hallmarks of T2D, leads to increased PFKFB3 expression. These results demonstrate a novel functional relationship between endothelial metabolism, ROS, and NO balance that may contribute to endothelial dysfunction in obesity.

## 1. Introduction

Over the past decades, the levels of obesity have risen alarmingly and it is one of the major health problems in developed and developing countries [1]. According to the World Health Organization (WHO), in 2022, one in eight people worldwide were living with obesity [2]. It is well established, that excessive body weight and obesity [3], are major risk factors for cardiovascular disease in both males and females [4,5], which in large part is due to an altered metabolic state [6,7].

One of the earliest features of cardiovascular dysfunction in obesity is the loss of endothelial function and impaired nitric oxide (NO) signaling [8]. NO is best known as the mediator of endothelium-dependent relaxation, but it is also vital for maintaining blood vessel homeostasis through the inhibition of leukocyte adhesion, platelet activation, and smooth muscle proliferation [9]. In blood vessels, NO is synthesized by endothelial nitric oxide synthase (eNOS), and dysregulation of eNOS has been shown to occur through changes in its expression and post-translational modifications, such as phosphorylation [9]. The biological actions of NO can be further compromised by rapid binding to reactive oxygen species (ROS), such as superoxide anions, which are generated by NADPH oxidases (NOX), as well as numerous other enzyme systems [10]. It has been previously shown that an imbalance between NO and superoxide-generating pathways is responsible for the loss of endothelial function in obese type 2 diabetic mice [11]. However, the signaling pathways in the endothelium of people with obesity that connect altered metabolism with loss of NO and increased superoxide production remain incompletely defined.

It is well established that type 2 diabetes (T2D) is associated with high blood glucose levels. Furthermore, endothelial cells are more susceptible to hyperglycemia-induced damage than other cell types [12] because of their ability to uptake glucose, as the primary glucose transporter in ECs is via the insulin-insensitive Glut-1 [13,14]. This is one mechanism distinguishing EC glucose metabolism from other cell types. Recently, a new hypothesis has emerged, suggesting that increased glucose metabolism in endothelial cells could be an essential driver of physiological/pathophysiological processes [15]. One of the main regulators of glycolysis in endothelial cells is the bi-functional enzyme 6-phosphofructo-2-kinase/fructose-2,6-bisphosphatase 3 (PFKFB3) [16]. Mechanistically, PFKFB3 catalyzes the synthesis (kinase activity) and degradation (phosphatase activity) of fructose-2,6-bisphosphate (F2,6P2), which is a highly potent allosteric activator of the rate-limiting enzyme of glycolysis, PFK1 [17]. Four isoforms of PFKFB (1–4) have been identified, and all of them encode a different version of the enzyme, 6-phosphofructokinase-2/fructose bisphosphatase-2 that produces and degrades F2,6P2. PFKFB1 is mainly expressed in hepatocytes and skeletal muscle and heart, PFKFB2 in the kidney, placenta, and pancreatic islets, PFKFB3 (also known as the inducible isoform) is ubiquitously expressed in vascular cell types, and PFKFB4 is expressed in testis [16]. Of these isoforms, PFKFB3 has the highest kinase activity with a 710:1 kinase/phosphatase ratio [18] and is a strong activator of glycolysis [16]. Inhibition of PFKFB3 suppresses glycolytic flux, indicating that it plays a major non-redundant role in regulating cellular glucose metabolism [19]. PFKFB3 gene expression is increased in cells with increased energy demands, such as rapidly proliferating cells [20,21], and several types of cancer [22]. Furthermore, PFKFB3 expression is increased in response to growth factors, such as insulin [23], inflammatory stimuli such as interleukin-6 (IL-6), LPS, interleukin-1β [24,25,26], and under hypoxic conditions via HIF-1α [24]. Although it is generally accepted that glycolysis occurs in the cytosol with very few exceptions (trypanosomes) [25], surprisingly, wild-type PFKFB3 (wt-PFKFB3) is predominantly localized in the nucleus [26], which is not seen with the other three PFKFB isoforms (PFKFB1-2, 4). In response to physiological increases in energy demands, AMPK can phosphorylate PFKFB3 at S461, leading to activation of the enzyme [27]. It has also been reported that the chemotherapeutic drug cisplatin induces the acetylation of PFKFB3 at its nuclear localization signal (NLS) at lysine 472, impairing the function of the NLS and resulting in the accumulation of PFKFB3 in the cytosol [28]. Nuclear PFKFB3, as opposed to cytosolic PFKFB3, regulates the expression of several cell cycle-related proteins, such as cyclin-dependent kinase 1 and Cdc25C, and decreases the expression of the cell cycle inhibitor p27 via a secondary effect of its metabolite, F2,6P2 [26], indicating a broader role for PFKFB3 than just the regulation of glycolysis.

We and others have previously shown that PFKFB3 expression is upregulated in the aortic endothelium and β-cells of type 1 diabetic (Akita) mice [29,30], in diabetic mouse liver [31], and in cardiac progenitor cells to promote insulin resistance in type 2 diabetic patients [32]. However, a direct link between obesity-induced EC dysfunction and PFKFB3 is not yet established. In the present study, we hypothesized that alterations in endothelial cell glucose metabolism contribute to impaired vascular relaxation through the dysregulation of eNOS signaling and decreased NO bioavailability. We further show that the upregulation of PFKFB3 is a hallmark of T2D in both micro- and macrovascular ECs, and we identify the signaling mechanisms upstream of eNOS by which glycolysis promotes endothelial dysfunction.

## 2. Materials and Methods

### 2.1. Cell Culture

Primary human aortic endothelial cells (HAECs) and type 2 diabetic human aortic endothelial cells (T2D HAECs) were obtained from LONZA (Walkersville, MD, USA) and cultured in endothelial cell basal medium 2 (EBM2), supplemented with endothelial cell growth medium-2 (EGM^TM^-2) BulletKit^TM^ medium (37 °C, 5% CO_2_). Cells were used for experiments between passages 2 and 6. To increase the expression of PFKFB3, HAECs were transduced with wild-type-PFKFB3 (wt-PFKFB3) adenovirus, K472/473A mutant PFKFB3 (cytosolic), and kinase-dead (KD) mutant-PFKFB3 expressing adenoviruses, or control adenoviruses expressing a green fluorescent protein (Ad-GFP), at a multiplicity of infection of 10–40 MOI in Opti-MEM media for 3 h. Next, media was replaced with complete EBM2, and 48 h later cells were collected, and samples were subjected to Western blot or real-time quantitative PCR (qPCR) analysis.

HEK293A and COS-7 cells were purchased from the American Type Culture Collection (ATCC, Manassas, VA, USA). HEK-eNOS and HEK-NOX5 cells, which constitutively express human eNOS or the NOX5 isoform, were generated using the Flp-in system^TM^ (ThermoFisher Scientific), as previously described. HEK293A, HEK-eNOS, HEK-NOX5, and COS-7 cells were cultured in Dulbecco’s modified Eagle’s medium (DMEM) containing 100 U/mL penicillin, 100 mg/mL streptomycin, and 10% fetal bovine serum (FBS), (37 °C, 5% CO_2_). To increase PFKFB3, cells were transfected using Lipofectamine 3000 (Invitrogen, Grand Island, NY, USA) according to the manufacturer’s instructions.

### 2.2. Animal Studies

All experiments involving mice were conducted under Augusta University Animal Care and Use Committee (Augusta, GA, USA; IACUC protocol number: 2011-0309) and in line with the US National Institutes of Health Guide for the Care and Use of Laboratory Animals. Leptin receptor mutant mice (*db*/*db*, #000697; Jackson Laboratory, Bar Harbor, ME, USA) were maintained on a C57/Bl6 background in the animal facility of Augusta University. Since *db*/*db* mice are sterile, progeny were generated from heterozygous mice. Heterozygous mice for leptin receptor mutation served as littermate controls. Homozygous mice develop obesity and T2D through overconsumption of food. A maximum of five sex-matched adult mice were housed in standard shoebox cages, ~75 in^2^, with irradiated Bed-o’Cob as bedding. Mice had ad libitum access to water (sterilized tap water) and food (Teklad 2918) and were kept under a 12/12-h dark/light cycle. Only male mice were used in the study at the age of 18–20 weeks. Mice had paper tubes to enrich their environment. Cages were changed every other week. Housing conditions and animal health were overseen by both the research team and the veterinary staff. Power analysis and prior experience were used to define the number of animals in each group to achieve statistical significance.

For experiments, mice were randomly selected for each group, and the primary endpoint was to determine the expression pattern of eNOS, NOX1, and PFKFB3 genes using qPCR analysis. Animals were excluded if they died prematurely due to any unexpected condition or technical errors. For each study, three different investigators were involved in executing the different stages of experiments, such as randomization, cell isolation, and molecular analysis. Animals were sacrificed at approximately 20 weeks of age by being anesthetized in an induction chamber with 5% isoflurane at 1 L/min O_2_ and decapitated by guillotine.

### 2.3. Endothelial Cell Isolation

Male mice were housed separately before the experiments. Conduit (aorta) and resistance (mesenteric) arteries were isolated from 4 to 6 euthanized mice. First, the arteries were flushed with sterile PBS and trimmed of any visceral adipose tissue. Isolated arteries were digested with diapase/collagenase II at 37 °C for 1 h, spun down, and resuspended in PBS. Suspensions were incubated with anti-CD31 microbeads (Miltenyi Biotec, Bergisch Gladbach, Germany; Lot no. 5191031675) at 4 °C for 20 min. Suspensions were then applied to a magnetic column, where flow-through fractions were collected, followed by elution fractions containing isolated CD31+ endothelial cells. Cells were then stored in Trizol or Laemmli sample buffer for qPCR or WB, respectively.

### 2.4. Gene Expression Analysis

RNA was isolated using Direct-zol RNA Miniprep Plus Kit (Zymo, Irvine, CA, USA; Lot no. ZRC204808) as per the manufacturer’s instructions. cDNA was synthesized using OneScript cDNA Synthesis SuperMix (ABM, New York, NY, USA; cat no. G452) as per the manufacturer’s instructions. qPCR was performed in a CFX-Connect Real-Time PCR Detection System (Bio-Rad, Hercules, CA, USA) with BrightGreen Express 2× qPCR MasterMix-iCycler (ABM). Briefly, genetic products were amplified using specific primer sets for each gene of interest (Table 1) at 95 °C for 3 min followed by 40 cycles of 95 °C for 15 s, 58.5 °C for 15 s, and 72 °C for 15 s. Gene expression was calculated using the 2^−ΔΔCT^ method, normalized to 18S rRNA or GAPDH as an internal control.

### 2.5. Site-Directed Mutagenesis

Site-directed mutagenesis was carried out using a Q5 Site-Directed Mutagenesis kit (New England Biolabs, Ipswich, MA, USA) according to the manufacturer’s instructions. The following primer pairs were used to produce the cytosolic and kinase-dead (kinase inactive) mutations, which were all verified by DNA sequencing: K472A/K473A mutant (cytosolic)was generated using the following primers: 5′-CCTCGCATCAACAGCTTTGAGGAGCATGTGGC-3′ (forward primer), and 5′-GGCTGCGGTGGGTTCGGGGCTGGC-3′ (reverse primer); R75A/R76A mutant (kinase-dead) was generated using the following primers: 5′-GGAGGCTGTGAAGCAGTACAGC-3′ (forward primer) and 5′-GCTGCATACTCCCCGACGTTGAA-3′ (reverse primer).

### 2.6. Measurement of Superoxide Production

HAECs were transduced with 10 MOI of Nox1 alone or with 10 MOI of Nox1, NoxA1, and NoxO1 adenoviruses, respectively, in the presence or absence of GFP, wt-, cyt-, or KD-PFKFB3 adenoviruses. In other experiments, HEK-NOX5 cells constitutively overexpressing human NOX5 were transfected with GFP, wt-, cyt-, or KD-PFKFB3 in pcDNA3.1. Forty-eight h later, transduced HAECs or transfected HEK-NOX5 cells were subcultured into 96-well white microplates at ∼10^4^ cells/well density and cultured overnight. Next, the cells were washed once with HBSS, and culture media were changed to phenol-free Dulbecco’s modified Eagle’s Medium (Sigma Aldrich, St. Louis, MO, USA) containing 400 µM L-012 (Wako Chemicals, Richmond, VA, USA) and 1 mM activated orthovanadate [33]. The cells were incubated for 10 min at 37 °C in a CO_2_ incubator; then, luminescence was recorded using a PolarSTAR luminometer (BMG Labtech, Cary, NC, USA), as previously described [33].

### 2.7. Measurement of Nitric Oxide Release

Nitric oxide production was measured by NO-specific ozone chemiluminescence using a Sievers NO Analyzer, NOA 280i (Sievers Instruments Inc., Boulder, CO, USA), as previously described [34]. HEK-eNOS cells overexpressing GFP, wt-, cyt-, or KD-PFKFB3 in pcDNA3.1, HAECs, or T2D HAECs, were cultured for 48 h in cell culture microplates, after which the media were collected for analysis of basal NO. In other experiments, the media were replaced with fresh complete media, supplemented with either vehicle (in control experiments) or 1 μM ionomycin for 30 min, and media were collected for subsequent analysis of stimulated NO release. During this period of incubation, NO that is released from cells into the media is primarily converted to nitrite in the presence of oxygen. Protein in the cell culture medium was precipitated with 100% ethanol in a 1:2 ratio. Twenty-five microliters of each sample were applied to the reaction chamber, where nitrite was converted to NO by sodium iodide and liberated by nitrogen purging. The NO liberated was converted by ozone to NO_2,_ and the chemiluminescence equivalent to NO formation was calculated using a standard curve.

### 2.8. Seahorse Assay

Primary human aortic endothelial cells (P3-6) were seeded at a cell density of 70,000 cells/well in Seahorse XFe24 cell culture plates (Agilent, Cat. #100777-004, Santa Clara, CA, USA) and cultured overnight at 37 °C in a CO_2_ incubator. The next day the cells were washed with Seahorse XF DMEM (pH 7.4) supplemented with 1 mM pyruvate, 2 mM glutamine, and 10 mM glucose. Then Seahorse XF96 glycolytic rate assays (Agilent, Catalogue # 103344-100, Santa Clara, CA, USA) were performed to evaluate the glycolytic rate according to the manufacturer’s instructions.

### 2.9. Immunoblotting Analysis

Cells were washed once with ice-cold PBS on ice, then lysed in 2× Laemmli Sample Buffer, scraped, sonicated for 3 × 10 s, and then boiled for 5 min at 100 °C. Protein samples were separated by 10% SDS-PAGE and transferred to a 0.20 µM pore size nitrocellulose membrane using the Trans-Blot^®^ Turbo™ Transfer System, 1.3 A and 25 V for 10 min. The membranes were then blocked with 5% (*w*/*v*) nonfat dry-milk powder solution in TBS-Tween 20 (TBST) and incubated overnight at 4 °C with specific antibodies (Table 2).

After incubation with the primary antibodies, the membranes were washed three times with TBST and incubated with horseradish peroxidase (HRP) conjugated secondary antibody. Immunoreactive proteins were visualized by enhanced chemiluminescence (ECL) using autoradiography films. Representative images were cropped using Adobe Photoshop Software (Adobe Systems Inc., San Jose, CA, USA). The brightness and contrast of the images were adjusted in a linear manner across the whole image in some instances. ImageJ software (Research Services Branch, National Institute of Health, Bethesda, MD, USA) was used for densitometry analyses.

### 2.10. Immunoprecipitation

HEK-eNOS cells were transfected with wt-PFKFB3 using Lipofectamine 3000™ transfection reagent (ThermoFisher Scientific Inc., Waltham, MA, USA) according to the manufacturer’s instructions. After 48 h of incubation, cells were lysed using Pierce™ IP Lysis Buffer (ThermoFisher Scientific Inc., Waltham, MA) containing 0.5% (*v*/*v*) protease inhibitor and centrifuged at 13,000× *g* for 10 min at 4 °C. Immunoprecipitation of eNOS was carried out using mouse anti-eNOS (BD Biosciences, Franklin Lakes, NJ, USA) antibody (mouse IgG was used as control) coupled with Pierce^TM^ protein A/G magnetic beads. After overnight incubation with the cell lysates at 4 °C, the beads were washed 3x with washing buffer consisting of 0.1% (*v*/*v*) Triton X-100, 0.5 mM NaCl, 50 mM Tris-HCl (pH 7.4), 20 mM EDTA, and 0.5% (*v*/*v*) protease inhibitor cocktail. Each of the samples was then boiled in 100 μL 2× Laemmli buffer for 5 min at 100 °C. Immune complexes were analyzed by immunoblotting.

### 2.11. Confocal Microscopy

COS7s were transfected with wild-type-, cytosolic-, or kinase-dead-PFKFB3 in the pcDNA3.1 plasmid using Lipofectamine 3000 transfection reagent and cultured at 37 °C, 5% CO_2_. After 48 h of incubation, the transfected cells were trypsinized, plated onto gelatin-coated glass coverslips, and grown overnight. Next, the cells were washed with HBSS and fixed with 4% (*v*/*v*) PFA for 10 minutes. Then, the cells were permeabilized with 0.1% (*v*/*v*) Triton X-100, 4% (*m*/*v*) BSA, and 0.01% (*m*/*v*) NaN3 in PBS (pH 7.4) for 1 h, washed three times with 4% (*m*/*v*) BSA in PBS, and once with antibody diluting buffer (0.1% (*v*/*v*) Triton X-100, 0.1% (*m*/*v*) BSA, and 0.01% (*m*/*v*) NaN3 in PBS (pH 7.5) for 10 min each time. After blocking, the cells were incubated with rabbit anti-PFKFB3 antibody at a 1:200 dilution in an antibody diluting buffer for 3 h at room temperature. Next, the cells were washed gently (three times) with PBS and incubated with goat polyclonal Alexa-488 (1:200)-conjugated secondary antibodies for 1 hour at room temperature. Finally, the cells were washed three times with PBS and covered in Prolong Gold antifade mounting medium. The localization of PFKFB3 mutants was visualized using a Zeiss LSM780 upright laser scanning confocal microscope (Zeiss, Oberkochen, Germany). The optical thickness of the images was 1 μm.

### 2.12. Cell Fractionation

HEK293 cells were transfected with GFP (control), wild-type-, cytosolic-, or kinase-dead PFKFB3 in pcDNA3.1 vector, using Lipofectamine 3000 transfection reagent and cultured for 48 h at 37 °C, 5% CO_2_, followed by cell fractionation using NE-PER nuclear and cytosolic extraction reagent (ThermoFisher Scientific Inc., Waltham, MA, USA) according to the manufacturer’s instructions. In short, after trypsinization, transfected HEK293 cells were collected, and washed with PBS. Then, 9 × 10^6^ cells were transferred from each group into new pre-chilled microcentrifuge tubes and centrifuged at 500× *g* for 3 min. The supernatant was removed, and 200 μL CER I buffer was added to 20 μL cell pellet. The pellet was vortexed for 15 s, and then 11 μL CER II was added. The cells were vortexed and after 1 min of incubation, the lysates were centrifuged for 5 min at maximum speed. The supernatants were immediately transferred into new microcentrifuge tubes, and 100 μL of NER buffer was added to the insoluble fraction containing the nuclei. The pellets were vortexed every 10 min for 40 min; the lysates were centrifuged again at maximum speed, 4 °C, and the supernatants were again immediately transferred into new tubes. The resulting cytosolic and nuclear fractions were then boiled in Laemmeli sample buffer for 5 min, and the samples were subjected to Western blot analysis.

### 2.13. Statistical Analysis

Statistical analyses were performed using GraphPad Prism 10 Software (GraphPad Software, Boston, MA, USA), and all data are expressed as means ± standard error of the mean (SEM). When two groups were compared, a student *t*-test was performed. Analyses between multiple groups were performed using one-way ANOVA with Tukey’s or Dunnet’s post hoc test where appropriate. Statistical significances were considered as *p* < 0.05.

## 3. Results

### 3.1. Type 2 Diabetes Results in Decreased eNOS Expression and NO Bioavailability and Correlates with Endothelial Glycolysis

**Figure 1:**
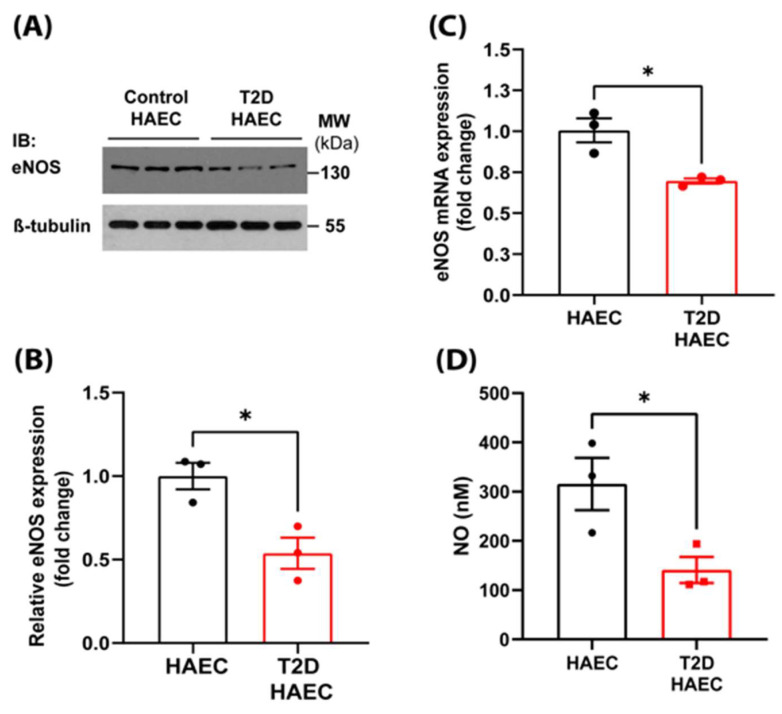
eNOS expression and NO bioavailability is decreased in type 2 diabetes. (**A**) Protein expression of eNOS and quantification of Western blots (**B**) in control and type 2 diabetic HAECs (n = 3, unpaired *t*-test). (**C**) Expression of eNOS mRNA in HAECs derived from control (healthy) or type 2 diabetic patients as determined by qRT-PCR (n = 3, unpaired *t*-test). (**D**) Nitric oxide (NO) production in control and type 2 diabetic HAECs was measured in cell cultures supernatant 48 h after sub-culturing by NO-specific chemiluminescence using a Sievers 280i NOA (n = 3, unpaired *t*-test). All data are represented as mean ± SE; * *p* < 0.05.

Nitric oxide is a critical vasodilatory mediator that is produced by eNOS in endothelial cells to maintain blood flow and pressure [35]. Decreased bioavailable NO has been reported in T2D patients, in large part due to altered production and scavenging by reactive oxygen species [36]. However, the expression of eNOS and its function have not been studied in endothelial cells derived directly from T2D patients before. To further investigate the mechanisms that could result in decreased NO production, we investigated eNOS expression at the transcriptional and translational levels and found a significant decrease in both eNOS protein (Figure 1A,B) and mRNA expression (Figure 1C) in endothelial cells derived from human aorta. Next, we investigated eNOS enzyme activity in cells and found that consistent with the transcriptional and translational changes in eNOS expression, NO production was also decreased in T2D endothelial cells compared to endothelial cells from control donors (Figure 1D).

**Figure 2:**
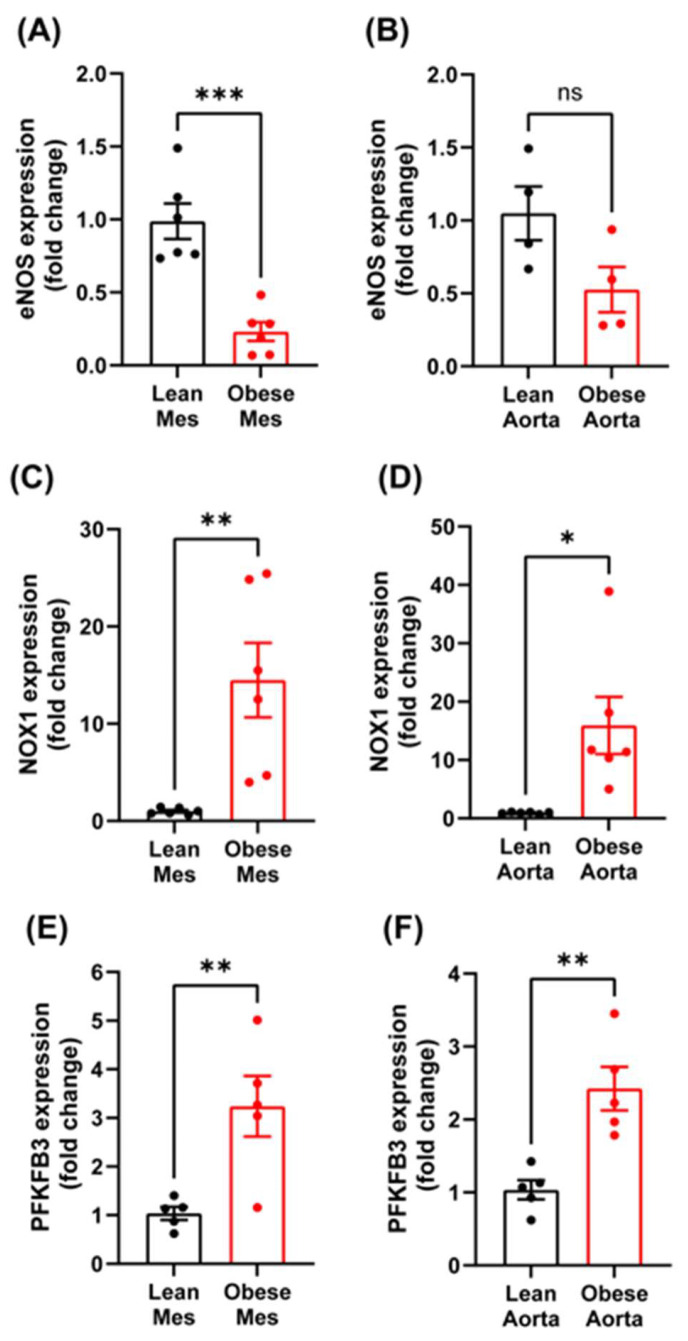
Characterization of differential gene expression patterns of eNOS, NOX1, and PFKFB3 in type 2 diabetic mouse micro- and macrovascular endothelial cells. (**A**) eNOS mRNA expression levels in mesenteric or (**B**) aortic endothelial cells. (**C**) NOX1 mRNA expression levels in mesenteric or (**D**) aortic endothelial cells. (**E**) PFKFB3 mRNA expression levels in mesenteric or (**F**) aortic endothelial cells. Data are represented as mean ± SE; ns = not-significant; Unpaired *t*-test, * *p* < 0.05, ** *p* < 0.01, *** *p* < 0.001.

To strengthen these data, we performed qPCR analysis on aortic and mesenteric artery ECs derived from healthy or diabetic mice with obesity (Figure 2A,B). Similarly to human data, eNOS expression was remarkably decreased in aortic ECs, and we observed a significant decrease in eNOS mRNA expression in the microvasculature of obese diabetic animals. Next, we investigated the expression of NOX1, a major source of ROS production in ECs that we and others have previously shown [11,37,38]. We found that NOX1 expression was highly upregulated in macro- and microvascular ECs of obese diabetic animals (Figure 2C,D). We next investigated whether endothelial cell glycolytic metabolism was altered in obese, hyperglycemic mice by assessing PFKFB3 expression, which is a rate-limiting step in glycolysis [39]. We found that PFKFB3 expression was significantly increased in both macro and microvascular ECs (Figure 2E,F), suggesting an increase in the rate of glycolysis.

**Figure 3:**
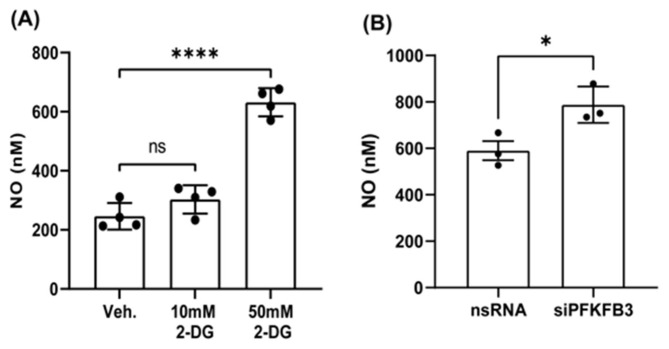
Endothelial glucose metabolism regulates nitric oxide production. (**A**) HEK-eNOS cells cultured in high-glucose-containing cell culture media were treated with vehicle (Veh.), 10 mM or 50 mM glucose analog 2-deoxy-D-glucose (2-DG) for 48 h and NO was measured from the cell cultures supernatant with ozone-chemiluminescence method. Data are represented as mean ± SE, (n = 4); One-way ANOVA with Tukey’s post hoc testing, ns = non-significant, **** *p* < 0.0001. (**B**) PFKFB3 silencing in HEK-eNOS cells. Media were collected 72 h post-silencing, and NO was analyzed as described above. Data are represented as mean ± SE, (n = 3); Student *t*-test, * *p* < 0.05.

Next, we assessed the functional significance of upregulated PFKFB3 gene expression, an indicator of increased glucose metabolism, on endothelial function. First, we measured NO levels in the presence and absence of the glycolysis inhibitor, 2DG (10–50 mM) (Figure 3A). Surprisingly, we found that the inhibition of glycolysis increased NO production in HAECs. We next investigated the direct role of PFKFB3 on NO production by silencing PFKFB3 in HEK-eNOS cells and found that NO levels were significantly elevated. Together, these data indicate that compromised endothelial function and NO bioavailability in T2D may be due, at least in part, to changes in endothelial metabolism and, more specifically, increased glycolysis.

### 3.2. PFKFB3 Induces Endothelial Cell Dysfunction via Altering eNOS Signaling

**Figure 4:**
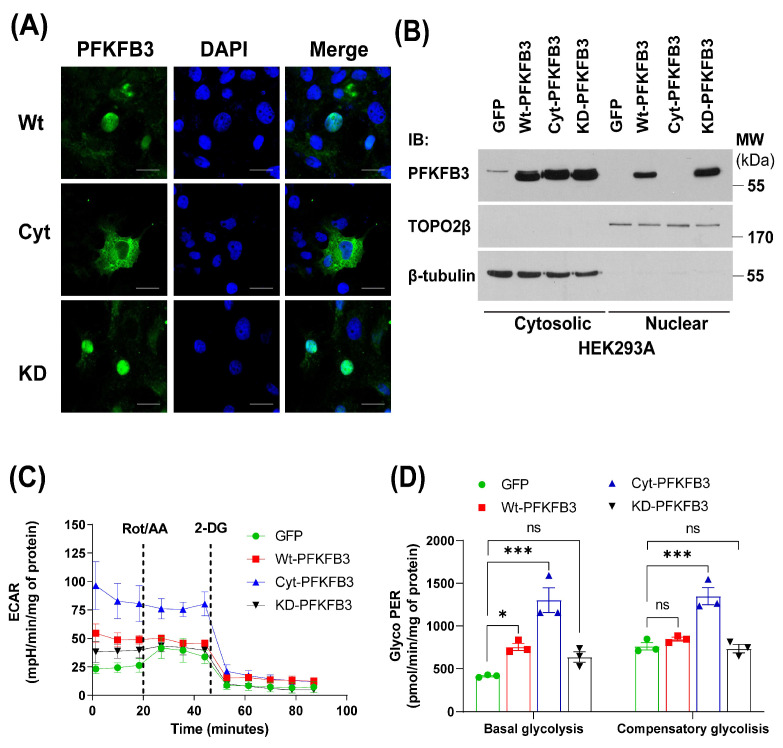
Characterization of mutant PFKFB3 constructs. (**A**) COS7 cells were transfected with wild-type (wt), cytosolic (cyt), or kinase-dead (KD) mutant PFKFB3, and their localization determined by confocal microscopy. Scale bar 25 μm. (**B**) Subcellular fractionation of HEK293A cells transfected with GFP, wt-, cyt-, or KD-PFKFB3. (**C**) Glycolytic rate assay measurement of HAECs transduced with GFP, wt-, cyt-, or Kinase dead PFKFB3 adenoviruses. Real-time ECAR values at basal level and after ROT/AA and 2-deoxy-D-glucose (2-DG) treatments were monitored by the Seahorse XFe24 analyzer. (**D**) Quantification of basal and compensatory glycolysis. Data are represented as mean ± SE, (n = 3); One-way ANOVA with Tukey’s post hoc testing, ns = non-significant, * *p* < 0.05, *** *p* < 0.001.

Although PFKFB3 has a predominant role as a rate-limiting step in regulating glycolytic metabolism in the cytosol, paradoxically, it is predominantly found in the nucleus. Yalcin et al., have demonstrated that wild-type PFKFB3 can regulate the expression of Cdk1, Cyclin D3, and Cdc25C, as well as p27 phosphorylation, indicating potential functional effects of nuclear-localized PFKFB3 on cell cycle regulation [26]. These data prompted us to generate a K472/473A (cytosolic, hereafter referred to as cyt-PFKFB3) and kinase-dead (R75/76A, hereafter referred to as KD-PFKFB3) mutants of PFKFB3. Next, we confirmed the subcellular localization of wild-type and mutant PFKFB3 isoforms by confocal microscopy (Figure 4A). Indeed, wt- and KD -PFKFB3 were localized to the nuclei of transfected cells, while cyt-PFKFB3 was excluded from the nucleus (middle panels). These data were confirmed using cell fractionation to isolate nuclear and cytosolic proteins (Figure 4B) revealing wt- and KD-PFKFB3 in both subcellular compartments and cyt-PFKFKB3 only in the cytosolic fraction. To investigate the impact of the above mutations and changes in subcellular location on the function of PFKFB3, we generated adenoviruses to overexpress wt-, cyt-, or KD-PFKFB3 or GFP (control) in HAECs and performed glycolytic rate analysis using a Seahorse XFe24 bio-analyzer (Figure 4C,D). Our data indicate that compared to control and KD-PFKFB3, both the wt- and cyt-PFKFB3 significantly increased the basal glycolytic rate of endothelial cells. Furthermore, cyt-PFKFB3 overexpressing HAECs have a significantly higher compensatory glycolytic rate, indicating increased glucose metabolism.

**Figure 5:**
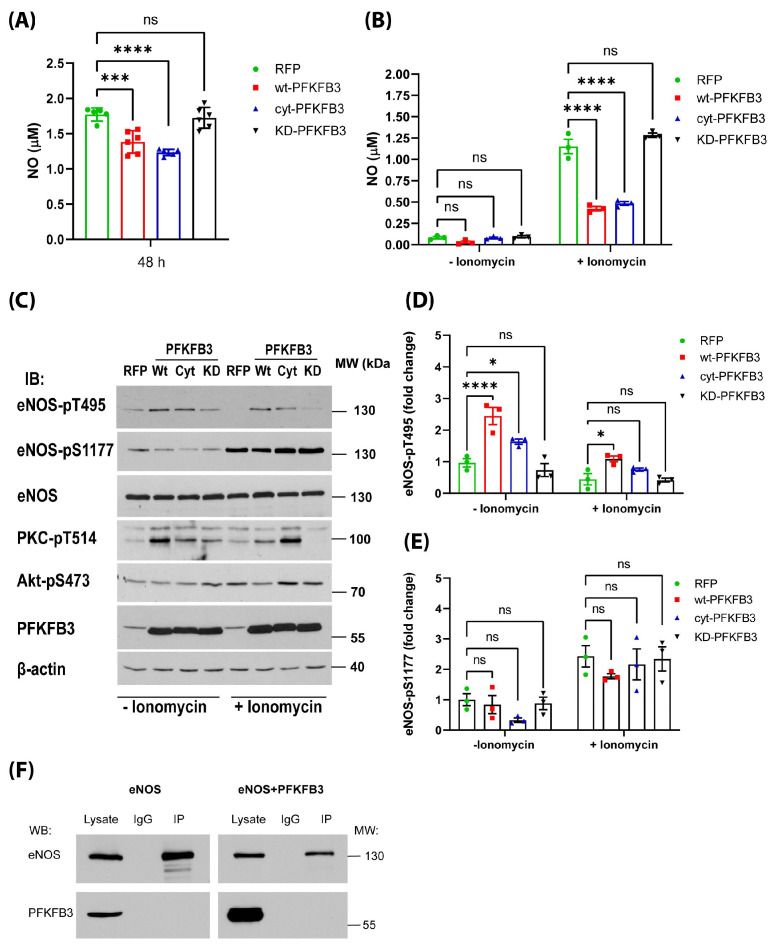
PFKFB3 kinase activity decreases nitric oxide and eNOS signaling. (**A**) Wt-, Cyt-, KD-PFKFB3, or RFP (control) were expressed in HEK-eNOS cells, and after 48 h of incubation NO levels were measured from cell cultures supernatant using a Sievers 280i NOA. (**B**) HEK-eNOS cells overexpressing wt-, cyt-, KD-PFKFB3, or RFP (control) were treated with vehicle (Veh.) or ionomycin (1 μM) for 30 min then NO was measured using NO-specific chemiluminescence. (**C**) Representative images of eNOS-T495, Ser1177, PKC-pT514, Akt-pSer473 phosphorylation, and PFKFB3 overexpression. (**D**,**E**) Quantification of Western blots (**C**) of eNOS pT495 and pS1177 levels, respectively. (**F**) Western blot analysis of eNOS binding partners in immunoprecipitates. In each lane lysates from HEK-eNOS cells (left panel) or from HEK-eNOS cells overexpressing wt PFKFB3 (right panel) were immunoprecipitated using anti-eNOS antibody or mouse IgG (control). Immunoprecipitated proteins were subjected to Western blotting with anti-eNOS and anti-PFKFB3 antibodies. All data are represented as mean ± SE, (n = 4); One-way ANOVA with Tukey’s post hoc testing, ns = non-significant, * *p* < 0.05, *** *p* < 0.001, **** *p* < 0.0001.

Since PFKFB3 can regulate processes other than glycolysis, such as protein expression and phosphorylation, in subsequent experiments, we have investigated the effect of these PFKFB3 constructs on NO production and eNOS post-translational modifications. We found that overexpression of both wt- and cyt-PFKFB3 in HEK-eNOS cells significantly decreased NO bioavailability over 48 h of incubation as opposed to GFP and KD-PFKFB3 overexpressing cells (Figure 5A). eNOS activity is regulated by changes in intracellular Ca^2+^ concentrations [40]. Therefore, we next assessed NO production following ionomycin stimulation, which increases intracellular Ca^2+^ levels. We found that in contrast to GFP and KD-PFKFB3-transfected cells wt-, and cyt-PFKFB3 both significantly attenuated ionomycin-stimulated increases in NO production (Figure 5B). Dysregulation of eNOS activity and NO production has been shown to occur not only through changes in its expression but via post-translational modifications [41,42,43]. Phosphorylation is one of the most important post-translational modifications regulating eNOS activity [34,44]. Thus, we next investigated eNOS phosphorylation at both inhibitory (T495) and activating (S1177) sites [45]. Interestingly, overexpression of wt-, and cyt-PFKFB3 significantly increased eNOS phosphorylation at T495 in parallel with an increase in pPKC-T514, which lies in the PKC activation loop. At the same time, pAkt-S473 levels did not change with WT or Cyt PFKFB3 (Figure 5C–E). The addition of ionomycin increased the eNOS-S1177 phosphorylation; however, phosphorylation levels of eNOS-T495 remained high, which could have contributed to the decreased NO levels. To investigate if changes in eNOS phosphorylation are due to elevated F2,6-BP levels or direct effects of PFKFB3, we immunoprecipitated eNOS from HEK-eNOS cells overexpressing wt-PFKFB3. However, we did not see a direct protein–protein interaction between eNOS and PFKFB3 despite the high intracellular levels of these proteins (Figure 5F).

### 3.3. Upregulation of PFKFB3 Expression Increases NOX Activity

**Figure 6:**
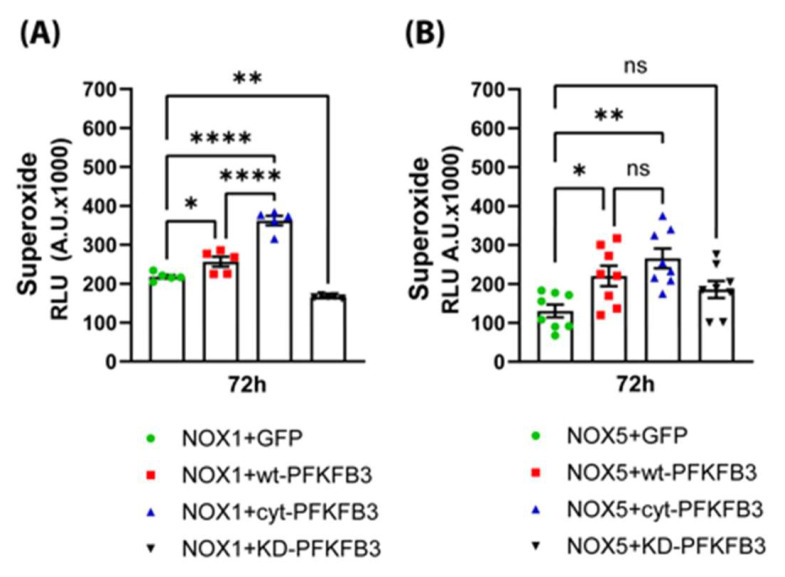
PFKFB3 increases the production of reactive oxygen species. (**A**) PFKFB3 increases NOX1 activity. HAECs were transduced with NOX1, (also with NOX activator 1 and NOX organizer 1 subunits) in the presence of GFP (control) or wt-, cyt- or KD-PFKFB3 adenoviruses and superoxide anion production was detected using enhanced L-012 chemiluminescence. (**B**) PFKFB3 increases NOX5 activity. HEK-NOX5 cells were transfected with GFP (control) or wt-, cyt- or KD-PFKFB3 constructs, and 72 h later superoxide production was detected using L-012 chemiluminescence. Data are represented as mean ± SE, (n = 5); One-way ANOVA with Dunnett’s post hoc testing; ns = non-significant, * *p* < 0.05, ** *p* < 0.01, **** *p* < 0.0001.

Reactive oxygen species generated by NADPH oxidases are known to contribute to vascular dysfunction in obesity [11,46]. To further investigate the mechanisms by which PFKFB3 contributes to endothelial dysfunction and decreased NO bioavailability, we transduced HAECs with NOX1 adenovirus (in addition to its coactivators NOXA1 and NOXO1) and either GFP (control) or wt-, cyt-, or KD-PFKFB3 (Figure 6A). We found that wt- and cyt-PFKFB3 significantly increased superoxide production; however, the ability of cyt-PFKFB3 to stimulate superoxide production was more pronounced. To investigate whether the effect of PFKFB3 on ROS production was unique to NOX1, we also investigated the effects of the above-mentioned PFKFB3s on NOX5 activity. NOX5 is different from other NOXs because it is the only calcium-activated isoform and does not require cytosolic subunits for its activity [46,47]. We found that superoxide production by NOX5 was similarly increased by wt- and cyt-PFKFB3 (Figure 6B).

### 3.4. High Glucose and TNFα Increase PFKFB3 Expression via an NFκB-Dependent Mechanism

**Figure 7:**
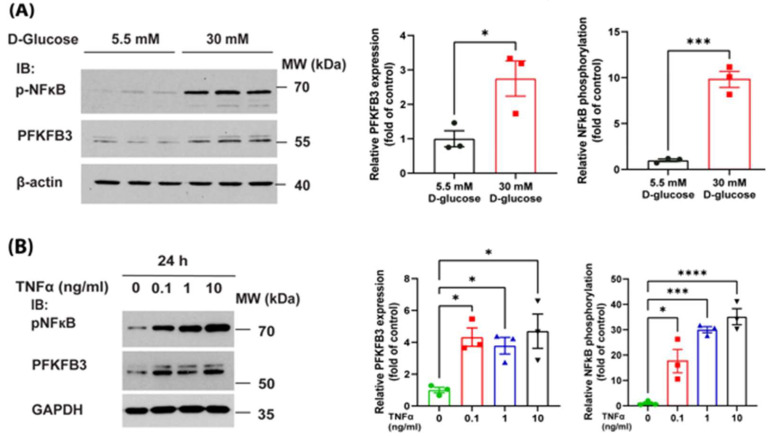
High glucose and TNFα increase PFKFB3 via a NFκB dependent mechanism. (**A**) Western blot analysis of the effect of high glucose conditions on PFKFB3 expression and phosphorylation of NFκB-p65. HAEC cells were cultured in normal glucose (5.5 mM) or high glucose (30 mM) containing complete media and phosphorylation of NFκBp65 and PFKFB3 expression was detected by Western blotting, shown in triplicates. Left panel: densitometry analysis of PFKFB3 expression and NFκBp65 phosphorylation. (**B**) Western blot analysis of the NFκB-p65 phosphorylation and PFKFB3 expression upon TNFα treatment. Left panel: densitometry analysis of PFKFB3 expression and NFκBp65 phosphorylation. All data are represented as mean ± SE, (n = 3); One-way ANOVA with Dunnett’s post hoc testing; ns = non-significant, * *p* < 0.05, *** *p* < 0.001, **** *p* < 0.0001.

It is well established that obesity and T2D are associated with increased blood glucose and TNFα levels, which may activate NFκB signaling [48]. Thus, we investigated the effect of high glucose and TNFα on PFKFB3 expression. Our data indicate that exposure of HAECs to high glucose (30 mM) significantly increased PFKFB3 expression, which was associated with increased pNFκB-p65 phosphorylation (Figure 7A). We also found that exposure of HAECs to TNFα increased pNFκB-p65 phosphorylation in a dose-dependent manner, and this was associated with an increase in PFKFB3 expression (Figure 7B).

## 4. Discussion

Endothelial cells, often collectively referred to as the endothelium, line the inner surface of all blood vessels. While the endothelium functions as a physical barrier between the blood and the surrounding tissues, it has many other roles in maintaining vascular homeostasis, including the regulation of blood flow and pressure [49]. Dysfunction of the endothelium contributes to almost all major diseases including T2D [50]. Several factors contribute to endothelial dysfunction in patients with obesity and T2D, such as hyperglycemia [51], insulin resistance [52], inflammation [53], advanced glycation end products (AGEs) [54], oxidative stress [11] and impaired NO bioavailability [10,55]. The endothelium is metabolically active, relying on glycolysis as its primary source of ATP (about 70%) [56]. Although it is well known that hyperglycemia can lead to increased glycolytic flux, elevated ROS, and decreased NO bioavailability [57,58], the exact mechanisms by which glycolysis regulates NO bioavailability have remained elusive. In the present study, we provide evidence that PFKFB3, the rate-limiting enzyme for glycolysis, contributes to decreased NO bioavailability via the promotion of eNOS phosphorylation at the inhibitory T495 site via a PKC-dependent mechanism. In addition, we provide evidence that increased PFKFB3 expression facilitates ROS production via NADPH oxidases. Lastly, we show that both increased glucose and TNFα levels contribute to the upregulation of PFKFB3 expression.

Loss of NO bioavailability can result from decreased production of NO and increased scavenging. In human endothelial cells in culture, we found that eNOS expression is decreased at the mRNA and protein levels in cells from patients with T2D. This was associated with decreased NO production. In support of these findings, we also found that eNOS mRNA expression is decreased in macro- and microvascular ECs from obese diabetic animals. Our data correlate with other in vitro studies where decreased NO production and eNOS uncoupling, as well as those reporting decreased eNOS protein levels, have been observed upon exposure to advanced glycation end products (AGEs) [57,59,60]. However, the reduced expression of eNOS we observed in isolated endothelial cells is not seen in all models of T2D and there are differences between cells in culture versus freshly isolated vessels [61]. Taken together, these findings provide support for the hypothesis that NO bioavailability can be impacted at the transcriptional, translational, and posttranslational levels in T2D.

The upregulation of PFKFB3 can enhance glycolytic flux, which is often observed in the pathogenic mechanisms underlying many diseases. The role of PFKFB3 in disease mechanisms is perhaps best characterized by the development of cancer [62], but increasing evidence supports the important roles of PFKFB3 in major vascular diseases, such as atherosclerosis [63], pulmonary hypertension [64], diabetic retinopathy [65], and angiogenesis [21]. Furthermore, as we and others have previously shown, PFKFB3 upregulation can be detected in conditions of metabolic stress, such as in type 1 diabetes in the endothelium [29] and in hepatocytes [31]. However, to the best of our knowledge, no previous study has reported PFKFB3 gene and protein upregulation in the context of endothelial dysfunction in ECs related to T2D. Here, we provide evidence for the first time that PFKFB3 is upregulated in T2D HAECs and that downregulation of PFKFB3 via siRNA silencing or inhibition of glycolysis using the substrate-based analog, 2-DG, increases the levels of NO. These data correlate with our previous findings, demonstrating that pharmacological inhibition of PFKFB3 with 3PO restored vascular function [29] and suggest that glycolysis, and more specifically, PFKFB3 itself, is a negative regulator of NO signaling. Siragusa et al., demonstrated that eNOS interacts with and inactivates pyruvate kinase 2 (PKM2), another rate-limiting enzyme of glycolysis, to decrease the glycolytic flux [66], and maintain endothelial redox homeostasis. These data indicate the existence of a more complicated relationship linking endothelial glycolytic metabolism and eNOS-derived NO, including a feedback mechanism between NO signaling and glycolysis.

The subcellular localization of PFKFB3 is unique among the four known PFKFB isoforms. Under baseline conditions, it is confined in the nucleus where it has been demonstrated to regulate cell-cycle dynamics [26]. Our results partially confirmed these previous findings, as we detected wt-PFKFB3 in the cytosolic compartment as well as in the nucleus. The relative expression of PFKFB3 in the nucleus versus cytosol is difficult to determine with precision but it is substantial. This raises questions about the functional role of nuclear partitioning of PFKFB3 given that the cells and cell lines used in our experiments have high glycolytic activity [67,68]. To study the mechanism by which PFKFB3 regulates eNOS activity and NO signaling, we used a cytosolic and kinase-inactive mutant of PFKFB3 that was characterized by Yalcin et al. (2009). These constructs proved to be useful in assessing whether PFKFB3 regulates eNOS and NO production via increased glycolysis in the cytosol or through actions in the nucleus. We found that both, cyt- and wt-PFKFB3 significantly increased the basal rate of glycolysis, although cyt-PFKFB3 to a much greater extent. However, they decreased NO production to a similar extent. Physiologically decreased NO levels could be achieved via eNOS post-translational modifications, such as phosphorylation via several kinases, which can increase or decrease eNOS activity. Studies have shown that PFKFB3 overexpression or silencing impacts Akt phosphorylation, [21], a well-described kinase regulating eNOS activation [43,45]. It has also been reported that hyperglycemia can impair eNOS phosphorylation at S1177 via o-GlcNAcylation [57]. However, we only detected a slight, but not significant, decrease in S1177 phosphorylation when wt- and cyt-PFKFB3 were overexpressed, with no changes in either Akt expression or phosphorylation. In contrast, both wt-, and cyt-PFKFB3 significantly increased the phosphorylation of the inhibitory T495 site on eNOS under basal conditions, which may explain the decreased NO levels. Surprisingly, the levels of peNOS-T495 were still elevated even when the cells were stimulated with ionomycin, which usually promotes dephosphorylation [69,70]. Our results indicate that increased PFKFB3 activity and the resulting stimulation of glycolytic flux maintain eNOS inhibitory phosphorylation even when stimuli that activate eNOS are applied, resulting in a remarkable decrease in NO production. The eNOS phosphorylation of eNOS on T495 has been shown to be due to PKC activity [71,72]. In our study, we demonstrated that pPKC-T514 was elevated when wt-, and cyt-PFKFB3 was overexpressed.

Obesity and T2D are closely associated with impaired endothelial function, which can result from heightened oxidative stress and increased pro-inflammatory cytokines [7]. Increased ROS result from enhancing NOX activity and have been demonstrated to reduce NO bioavailability and compromise endothelial function [73,74]. Therefore, we hypothesized that cyt-PFKFB3 upregulation and the accompanying increase in glycolytic flux can increase ROS production via the activation of NOXs, such as NOX1 and NOX5 [29,75], which, in turn, contribute to a decreased NO. Indeed, both wt- and cyt-PFKFB3 significantly enhanced NOX1 and NOX5 activity despite the functional differences between these NOXs. However, superoxide production was approximately 40% higher in cells expressing cyt-PFKFB3 versus wt-PFKFB3, which correlates with the higher basal glycolytic rates seen with the cyt-PFKFB3. However, the exact mechanisms by which PFKFB3 and glycolytic metabolism stimulate NOX activity remain unknown and the subject of future studies.

It is well known that a complex interplay of factors contributes to the pathophysiology of endothelial dysfunction in T2D, including hyperglycemia, as well as inflammatory cytokines, such as TNF-α [76]. While earlier studies in TNFα-receptor-deficient mice failed to support the concept that TNFα is a major contributor to obesity-associated insulin resistance, [77] increasing evidence supports a positive correlation between TNF-α-induced insulin resistance, NF-κB signaling, and pathogenesis of T2DM [48,78,79,80]. Others have shown that NF-κB signaling is increased in patients with obesity and T2D, and could not be reversed by exercise, suggesting refractory inflammation in these patients. The effect of hyperglycemia on endothelial cell signaling is also complex and controversial. Some have shown that chronic activation of the NF-κB pathway by hyperglycemia combined with ischemia results in increased IκBα levels, leading to the inactivation of the canonical NF-κB signaling pathway in PAD [81]. However, the general consensus is that hyperglycemia and low-grade inflammation contribute not only to NF-κB activation in T2D [82,83] but also endothelial dysfunction [84]. In accordance with these findings, we found that both high glucose levels and TNFα activate NFκB-p65 and upregulate PFKFB3. Thus, it is likely that together, they contribute to endothelial dysfunction in obesity-associated T2D.

Herein, we have used both in vivo and in vitro models, including a T2D animal model and endothelial cells derived from T2D patients, to study the effect of glucose metabolism on endothelial function, which is a strength of the presented work. However, there are some limitations to our studies. The activity of eNOS can be compromised by multiple mechanisms, namely by altered signaling mechanisms and protein expression. When analyzing the post-translational modifications of eNOS, we did not see a decrease in eNOS protein level upon 48 h of overexpression of either wt-, or cyt-PFKFB3, unlike that observed in T2D HAECs. This could be partially explained by the differences between our in vitro experimental conditions and the pathogenesis of T2D in vivo. Indeed, previous findings suggest that a loss in eNOS expression and NO production during the pathogenesis of T2D may take as long as 5 years after the onset of T2D [85]. This protracted mechanism may provide an explanation for the variation in previous findings in animal models, where the effectiveness of correcting poor glycemic control in diabetic rats was time dependent. The longer the duration of diabetes, the less likely it was to be able to reverse the declining NO levels [86]. These data suggest that the decline in eNOS expression in vivo and its role in causing endothelial dysfunction can be a chronic and progressive process in the pathogenesis of T2D.

## 5. Conclusions

The studies herein provide evidence that eNOS expression and activity are downregulated in endothelial cells in T2D. A major mechanism was due to increased glycolytic flux via PFKFB3, which alters the NO/ROS balance by facilitating the inhibitory phosphorylation of eNOS by PKC and increasing the activity of NOXs. Thus, pharmacological inhibition of PFKFB3 and appropriate glycemic control may be of therapeutic value in managing obesity-induced endothelial dysfunction. However, determining whether these interventions can reverse the loss of eNOS expression and activity in T2D requires further investigation.

## Figures and Tables

**Table 1:** List of primers.

Gene	Primer Pairs
Human eNOS	Forward primer 5′-TGATGGCGAAGCGAGTGAAG-3′ Reverse primer 5′-ACTCATCCATACACAGGACCC-3′
Mouse eNOS	Forward primer 5′-TCAGCCATCACAGTGTTCCC-3′ Reverse primer 5′-ATAGCCCGCATAGCGTATCAG-3′
Mouse NOX1	Forward primer 5′-CCTGATTCCTGTGTGTCGAAA-3′ Reverse primer 5′-TTGGCTTCTTCTGTAGCGTTC-3′
Mouse PFKFB3	Forward primer 5′-CAACTCCCCAACCGTGATTGT-3′ Reverse primer 5′-TGAGGTAGCGAGTCAGCTTCT-3′

**Table 2:** List of antibodies.

Antibody	Dilution Rate	Vendor	Cat. No.	RRID
eNOS	1:2000	Transduction Laboratories	610286	AB_2314377
eNOS-pS1177	1:1000	Cell Signaling Technology	9571	AB_329837
eNOS-pT495	1:1000	Cell Signaling Technology	9574	AB_2153176
PFKFB3	1:1000	Cell Signaling Technology	13123	AB_2617178
β-actin	1:1000	Cell Signaling Technology	5125	AB_1903890
β-tubulin	1:1000	Cell Signaling Technology	86298	AB_2715541
GAPDH	1:1000	Cell Signaling Technology	97166	AB_2756824
Akt-pS473	1:1000	Cell Signaling Technology	9271	AB_329825
PKC-pT514	1:1000	Cell Signaling Technology	38938	AB_2799144
NF-kappaB-p65-pS536	1:1000	Cell Signaling Technology	3031	AB_330559
TOPO2β	1:1000	Thermo Fisher Scientific	A300-950A	AB_805860

## Data Availability

The raw data supporting the conclusions of this article will be made available by the authors on request.

## References

[1] Sattar N., Neeland I.J., McGuire D.K. (2024). Obesity and Cardiovascular Disease: A New Dawn. Circulation.

[2] WHO Obesity and Overweight. https://www.who.int/news-room/fact-sheets/detail/obesity-and-overweight.

[3] Astrup A., Finer N. (2000). Redefining Type 2 diabetes: ‘Diabesity’ or ‘Obesity Dependent Diabetes Mellitus’?. Obes. Rev..

[4] (2017). Health Effects of Overweight and Obesity in 195 Countries over 25 Years. N. Engl. J. Med..

[5] Wilson P.W., D’Agostino R.B., Sullivan L., Parise H., Kannel W.B. (2002). Overweight and obesity as determinants of cardiovascular risk: The Framingham experience. Arch. Intern. Med..

[6] Lavie C.J., Milani R.V., Ventura H.O. (2009). Obesity and cardiovascular disease: Risk factor, paradox, and impact of weight loss. J. Am. Coll. Cardiol..

[7] Poirier P., Giles T.D., Bray G.A., Hong Y., Stern J.S., Pi-Sunyer F.X., Eckel R.H. (2006). Obesity and cardiovascular disease: Pathophysiology, evaluation, and effect of weight loss: An update of the 1997 American Heart Association Scientific Statement on Obesity and Heart Disease from the Obesity Committee of the Council on Nutrition, Physical Activity, and Metabolism. Circulation.

[8] Arcaro G., Zamboni M., Rossi L., Turcato E., Covi G., Armellini F., Bosello O., Lechi A. (1999). Body fat distribution predicts the degree of endothelial dysfunction in uncomplicated obesity. Int. J. Obes. Relat. Metab. Disord. J. Int. Assoc. Study Obes..

[9] Förstermann U., Sessa W.C. (2012). Nitric oxide synthases: Regulation and function. Eur. Heart J..

[10] Cai H., Harrison D.G. (2000). Endothelial Dysfunction in Cardiovascular Diseases: The Role of Oxidant Stress. Circ. Res..

[11] Thompson J.A., Larion S., Mintz J.D., Belin de Chantemèle E.J., Fulton D.J., Stepp D.W. (2017). Genetic Deletion of NADPH Oxidase 1 Rescues Microvascular Function in Mice with Metabolic Disease. Circ. Res..

[12] Salvatore T., Galiero R., Caturano A., Vetrano E., Loffredo G., Rinaldi L., Catalini C., Gjeloshi K., Albanese G., Di Martino A. (2022). Coronary Microvascular Dysfunction in Diabetes Mellitus: Pathogenetic Mechanisms and Potential Therapeutic Options. Biomedicines.

[13] Kaiser N., Sasson S., Feener E.P., Boukobza-Vardi N., Higashi S., Moller D.E., Davidheiser S., Przybylski R.J., King G.L. (1993). Differential Regulation of Glucose Transport and Transporters by Glucose in Vascular Endothelial and Smooth Muscle Cells. Diabetes.

[14] Veys K., Fan Z., Ghobrial M., Bouché A., García-Caballero M., Vriens K., Conchinha N.V., Seuwen A., Schlegel F., Gorski T. (2020). Role of the GLUT1 Glucose Transporter in Postnatal CNS Angiogenesis and Blood-Brain Barrier Integrity. Circ. Res..

[15] Leung S.W.S., Shi Y. (2022). The glycolytic process in endothelial cells and its implications. Acta Pharmacol. Sin..

[16] Okar D.A., Manzano A., Navarro-Sabatè A., Riera L., Bartrons R., Lange A.J. (2001). PFK-2/FBPase-2: Maker and breaker of the essential biofactor fructose-2,6-bisphosphate. Trends Biochem. Sci..

[17] Van Schaftingen E., Jett M.F., Hue L., Hers H.G. (1981). Control of liver 6-phosphofructokinase by fructose 2,6-bisphosphate and other effectors. Proc. Natl. Acad. Sci. USA.

[18] Sakakibara R., Kato M., Okamura N., Nakagawa T., Komada Y., Tominaga N., Shimojo M., Fukasawa M. (1997). Characterization of a human placental fructose-6-phosphate, 2-kinase/fructose-2,6-bisphosphatase. J. Biochem..

[19] Lu L., Chen Y., Zhu Y. (2017). The molecular basis of targeting PFKFB3 as a therapeutic strategy against cancer. Oncotarget.

[20] Chesney J., Mitchell R., Benigni F., Bacher M., Spiegel L., Al-Abed Y., Han J.H., Metz C., Bucala R. (1999). An inducible gene product for 6-phosphofructo-2-kinase with an AU-rich instability element: Role in tumor cell glycolysis and the Warburg effect. Proc. Natl. Acad. Sci. USA.

[21] Xu Y., An X., Guo X., Habtetsion T.G., Wang Y., Xu X., Kandala S., Li Q., Li H., Zhang C. (2014). Endothelial PFKFB3 Plays a Critical Role in Angiogenesis. Arterioscler. Thromb. Vasc. Biol..

[22] Da Q., Huang L., Huang C., Chen Z., Jiang Z., Huang F., Shen T., Sun L., Yan Z., Ye X. (2023). Glycolytic regulatory enzyme PFKFB3 as a prognostic and tumor microenvironment biomarker in human cancers. Aging.

[23] Riera L.s., Manzano A., Navarro-Sabaté A., Perales J.C., Bartrons R. (2002). Insulin induces PFKFB3 gene expression in HT29 human colon adenocarcinoma cells. Biochim. Biophys. Acta (BBA) Mol. Cell Res..

[24] Minchenko A., Leshchinsky I., Opentanova I., Sang N., Srinivas V., Armstead V., Caro J. (2002). Hypoxia-inducible factor-1-mediated expression of the 6-phosphofructo-2-kinase/fructose-2,6-bisphosphatase-3 (PFKFB3) gene. Its possible role in the Warburg effect. J. Biol. Chem..

[25] Río Bártulos C., Rogers M.B., Williams T.A., Gentekaki E., Brinkmann H., Cerff R., Liaud M.F., Hehl A.B., Yarlett N.R., Gruber A. (2018). Mitochondrial Glycolysis in a Major Lineage of Eukaryotes. Genome Biol. Evol..

[26] Yalcin A., Clem B.F., Simmons A., Lane A., Nelson K., Clem A.L., Brock E., Siow D., Wattenberg B., Telang S. (2009). Nuclear targeting of 6-phosphofructo-2-kinase (PFKFB3) increases proliferation via cyclin-dependent kinases. J. Biol. Chem..

[27] Marsin A.S., Bouzin C., Bertrand L., Hue L. (2002). The stimulation of glycolysis by hypoxia in activated monocytes is mediated by AMP-activated protein kinase and inducible 6-phosphofructo-2-kinase. J. Biol. Chem..

[28] Li F.L., Liu J.P., Bao R.X., Yan G., Feng X., Xu Y.P., Sun Y.P., Yan W., Ling Z.Q., Xiong Y. (2018). Acetylation accumulates PFKFB3 in cytoplasm to promote glycolysis and protects cells from cisplatin-induced apoptosis. Nat. Commun..

[29] Atawia R.T., Batori R.K., Jordan C.R., Kennard S., Antonova G., Bruder-Nascimento T., Mehta V., Saeed M.I., Patel V.S., Fukai T. (2023). Type 1 Diabetes Impairs Endothelium-Dependent Relaxation Via Increasing Endothelial Cell Glycolysis Through Advanced Glycation End Products, PFKFB3, and Nox1-Mediated Mechanisms. Hypertension.

[30] Nomoto H., Pei L., Montemurro C., Rosenberger M., Furterer A., Coppola G., Nadel B., Pellegrini M., Gurlo T., Butler P.C. (2020). Activation of the HIF1α/PFKFB3 stress response pathway in beta cells in type 1 diabetes. Diabetologia.

[31] Duran J., Obach M., Navarro-Sabate A., Manzano A., Gómez M., Rosa J.L., Ventura F., Perales J.C., Bartrons R. (2009). Pfkfb3 is transcriptionally upregulated in diabetic mouse liver through proliferative signals. FEBS J..

[32] Salabei J.K., Lorkiewicz P.K., Mehra P., Gibb A.A., Haberzettl P., Hong K.U., Wei X., Zhang X., Li Q., Wysoczynski M. (2016). Type 2 Diabetes Dysregulates Glucose Metabolism in Cardiac Progenitor Cells. J. Biol. Chem..

[33] Haigh S., Brown Z.L., Shivers M.A., Sellers H.G., West M.A., Barman S.A., Stepp D.W., Csanyi G., Fulton D.J.R. (2023). A Reappraisal of the Utility of L-012 to Measure Superoxide from Biologically Relevant Sources. Antioxidants.

[34] Church J.E., Fulton D. (2006). Differences in eNOS activity because of subcellular localization are dictated by phosphorylation state rather than the local calcium environment. J. Biol. Chem..

[35] Kelm M. (1999). Nitric oxide metabolism and breakdown. Biochim. Biophys. Acta (BBA) Bioenerg..

[36] Paneni F., Beckman J.A., Creager M.A., Cosentino F. (2013). Diabetes and vascular disease: Pathophysiology, clinical consequences, and medical therapy: Part I. Eur. Heart J..

[37] Padgett C.A., Bátori R.K., Speese A.C., Rosewater C.L., Bush W.B., Derella C.C., Haigh S.B., Sellers H.G., Corley Z.L., West M.A. (2023). Galectin-3 Mediates Vascular Dysfunction in Obesity by Regulating NADPH Oxidase 1. bioRxiv.

[38] Gray S.P., Di Marco E., Okabe J., Szyndralewiez C., Heitz F., Montezano A.C., de Haan J.B., Koulis C., El-Osta A., Andrews K.L. (2013). NADPH Oxidase 1 Plays a Key Role in Diabetes Mellitus–Accelerated Atherosclerosis. Circulation.

[39] Zuo J., Tang J., Lu M., Zhou Z., Li Y., Tian H., Liu E., Gao B., Liu T., Shao P. (2021). Glycolysis Rate-Limiting Enzymes: Novel Potential Regulators of Rheumatoid Arthritis Pathogenesis. Front. Immunol..

[40] Moncada S., Higgs A. (1993). The L-arginine-nitric oxide pathway. N. Engl. J. Med..

[41] Qian J., Fulton D. (2013). Post-translational regulation of endothelial nitric oxide synthase in vascular endothelium. Front. Physiol..

[42] Garcia V., Sessa W.C. (2019). Endothelial NOS: Perspective and recent developments. Br. J. Pharmacol..

[43] Fulton D., Gratton J.P., McCabe T.J., Fontana J., Fujio Y., Walsh K., Franke T.F., Papapetropoulos A., Sessa W.C. (1999). Regulation of endothelium-derived nitric oxide production by the protein kinase Akt. Nature.

[44] Fulton D., Gratton J.P., Sessa W.C. (2001). Post-translational control of endothelial nitric oxide synthase: Why isn’t calcium/calmodulin enough?. J. Pharmacol. Exp. Ther..

[45] Dimmeler S., Fleming I., Fisslthaler B., Hermann C., Busse R., Zeiher A.M. (1999). Activation of nitric oxide synthase in endothelial cells by Akt-dependent phosphorylation. Nature.

[46] DeVallance E., Li Y., Jurczak M.J., Cifuentes-Pagano E., Pagano P.J. (2019). The Role of NADPH Oxidases in the Etiology of Obesity and Metabolic Syndrome: Contribution of Individual Isoforms and Cell Biology. Antioxid. Redox Signal..

[47] Fulton D.J.R. (2019). The Molecular Regulation and Functional Roles of NOX5. Methods Mol. Biol..

[48] Alzamil H. (2020). Elevated Serum TNF-α Is Related to Obesity in Type 2 Diabetes Mellitus and Is Associated with Glycemic Control and Insulin Resistance. J. Obes..

[49] Aird W.C. (2008). Endothelium in health and disease. Pharmacol. Rep..

[50] Rajendran P., Rengarajan T., Thangavel J., Nishigaki Y., Sakthisekaran D., Sethi G., Nishigaki I. (2013). The vascular endothelium and human diseases. Int. J. Biol. Sci..

[51] Martin B.C., Warram J.H., Krolewski A.S., Bergman R.N., Soeldner J.S., Kahn C.R. (1992). Role of glucose and insulin resistance in development of type 2 diabetes mellitus: Results of a 25-year follow-up study. Lancet.

[52] Taylor R. (2012). Insulin resistance and type 2 diabetes. Diabetes.

[53] Akash M.S.H., Rehman K., Liaqat A. (2018). Tumor Necrosis Factor-Alpha: Role in Development of Insulin Resistance and Pathogenesis of Type 2 Diabetes Mellitus. J. Cell. Biochem..

[54] Khalid M., Petroianu G., Adem A. (2022). Advanced Glycation End Products and Diabetes Mellitus: Mechanisms and Perspectives. Biomolecules.

[55] Meza C.A., La Favor J.D., Kim D.-H., Hickner R.C. (2019). Endothelial Dysfunction: Is There a Hyperglycemia-Induced Imbalance of NOX and NOS?. Int. J. Mol. Sci..

[56] Culic O., Gruwel M.L., Schrader J. (1997). Energy turnover of vascular endothelial cells. Am. J. Physiol..

[57] Du X.L., Edelstein D., Dimmeler S., Ju Q., Sui C., Brownlee M. (2001). Hyperglycemia inhibits endothelial nitric oxide synthase activity by posttranslational modification at the Akt site. J. Clin. Investig..

[58] An Y., Xu B.T., Wan S.R., Ma X.M., Long Y., Xu Y., Jiang Z.Z. (2023). The role of oxidative stress in diabetes mellitus-induced vascular endothelial dysfunction. Cardiovasc. Diabetol..

[59] Thum T., Fraccarollo D., Schultheiss M., Froese S., Galuppo P., Widder J.D., Tsikas D., Ertl G., Bauersachs J. (2007). Endothelial Nitric Oxide Synthase Uncoupling Impairs Endothelial Progenitor Cell Mobilization and Function in Diabetes. Diabetes.

[60] Ren X., Ren L., Wei Q., Shao H., Chen L., Liu N. (2017). Advanced glycation end-products decreases expression of endothelial nitric oxide synthase through oxidative stress in human coronary artery endothelial cells. Cardiovasc. Diabetol..

[61] Fulton D., Harris M.B., Kemp B.E., Venema R.C., Marrero M.B., Stepp D.W. (2004). Insulin resistance does not diminish eNOS expression, phosphorylation, or binding to HSP-90. Am. J. Physiol. Heart Circ. Physiol..

[62] Shi L., Pan H., Liu Z., Xie J., Han W. (2017). Roles of PFKFB3 in cancer. Signal Transduct. Target Ther..

[63] Guo S., Li A., Fu X., Li Z., Cao K., Song M., Huang S., Li Z., Yan J., Wang L. (2022). Gene-dosage effect of Pfkfb3 on monocyte/macrophage biology in atherosclerosis. Br. J. Pharmacol..

[64] Cao Y., Zhang X., Wang L., Yang Q., Ma Q., Xu J., Wang J., Kovacs L., Ayon R.J., Liu Z. (2019). PFKFB3-mediated endothelial glycolysis promotes pulmonary hypertension. Proc. Natl. Acad. Sci. USA.

[65] Min J., Zeng T., Roux M., Lazar D., Chen L., Tudzarova S. (2021). The Role of HIF1α-PFKFB3 Pathway in Diabetic Retinopathy. J. Clin. Endocrinol. Metab..

[66] Siragusa M., Thöle J., Bibli S.I., Luck B., Loot A.E., de Silva K., Wittig I., Heidler J., Stingl H., Randriamboavonjy V. (2019). Nitric oxide maintains endothelial redox homeostasis through PKM2 inhibition. EMBO J..

[67] Henry O., Jolicoeur M., Kamen A. (2011). Unraveling the metabolism of HEK-293 cells using lactate isotopomer analysis. Bioprocess Biosyst. Eng..

[68] Fehr M., Lalonde S., Ehrhardt D.W., Frommer W.B. (2004). Live Imaging of Glucose Homeostasis in Nuclei of COS-7 Cells. J. Fluoresc..

[69] Fleming I., Fisslthaler B., Dimmeler S., Kemp B.E., Busse R. (2001). Phosphorylation of Thr(495) regulates Ca^2+^/calmodulin-dependent endothelial nitric oxide synthase activity. Circ. Res..

[70] Harris M.B., Ju H., Venema V.J., Liang H., Zou R., Michell B.J., Chen Z.P., Kemp B.E., Venema R.C. (2001). Reciprocal phosphorylation and regulation of endothelial nitric-oxide synthase in response to bradykinin stimulation. J. Biol. Chem..

[71] Chen F., Kumar S., Yu Y., Aggarwal S., Gross C., Wang Y., Chakraborty T., Verin A.D., Catravas J.D., Lucas R. (2014). PKC-dependent phosphorylation of eNOS at T495 regulates eNOS coupling and endothelial barrier function in response to G+ -toxins. PLoS ONE.

[72] Michell B.J., Chen Z.-p., Tiganis T., Stapleton D., Katsis F., Power D.A., Sim A.T., Kemp B.E. (2001). Coordinated Control of Endothelial Nitric-oxide Synthase Phosphorylation by Protein Kinase C and the cAMP-dependent Protein Kinase. J. Biol. Chem..

[73] Tan Y., Cheong M.S., Cheang W.S. (2022). Roles of Reactive Oxygen Species in Vascular Complications of Diabetes: Therapeutic Properties of Medicinal Plants and Food. Oxygen.

[74] Beckman J.S., Beckman T.W., Chen J., Marshall P.A., Freeman B.A. (1990). Apparent hydroxyl radical production by peroxynitrite: Implications for endothelial injury from nitric oxide and superoxide. Proc. Natl. Acad. Sci. USA.

[75] Qian J., Chen F., Kovalenkov Y., Pandey D., Moseley M.A., Foster M.W., Black S.M., Venema R.C., Stepp D.W., Fulton D.J. (2012). Nitric oxide reduces NADPH oxidase 5 (Nox5) activity by reversible S-nitrosylation. Free Radic. Biol. Med..

[76] Galicia-Garcia U., Benito-Vicente A., Jebari S., Larrea-Sebal A., Siddiqi H., Uribe K.B., Ostolaza H., Martín C. (2020). Pathophysiology of Type 2 Diabetes Mellitus. Int. J. Mol. Sci..

[77] Schreyer S.A., Chua S.C., LeBoeuf R.C. (1998). Obesity and diabetes in TNF-alpha receptor- deficient mice. J. Clin. Investig..

[78] Chen Y.-l., Qiao Y.-c., Xu Y., Ling W., Pan Y.-h., Huang Y.-c., Geng L.-j., Zhao H.-l., Zhang X.-x. (2017). Serum TNF-α concentrations in type 2 diabetes mellitus patients and diabetic nephropathy patients: A systematic review and meta-analysis. Immunol. Lett..

[79] El Sheikh W.M., Alahmar I.E., Salem G.M., El-Sheikh M.A. (2019). Tumor necrosis factor alpha in peripheral neuropathy in type 2 diabetes mellitus. Egypt. J. Neurol. Psychiatry Neurosurg..

[80] Mirza S., Hossain M., Mathews C., Martinez P., Pino P., Gay J.L., Rentfro A., McCormick J.B., Fisher-Hoch S.P. (2012). Type 2-diabetes is associated with elevated levels of TNF-alpha, IL-6 and adiponectin and low levels of leptin in a population of Mexican Americans: A cross-sectional study. Cytokine.

[81] Singh M.V., Wong T., Moorjani S., Mani A.M., Dokun A.O. (2024). Novel components in the nuclear factor-kappa B (NF-kappaB) signaling pathways of endothelial cells under hyperglycemic-ischemic conditions. Front. Cardiovasc. Med..

[82] Catrysse L., van Loo G. (2017). Inflammation and the Metabolic Syndrome: The Tissue-Specific Functions of NF-κB. Trends Cell Biol..

[83] Meyerovich K., Ortis F., Cardozo A.K. (2018). The non-canonical NF-κB pathway and its contribution to β-cell failure in diabetes. J. Mol. Endocrinol..

[84] Suryavanshi S.V., Kulkarni Y.A. (2017). NF-κβ: A Potential Target in the Management of Vascular Complications of Diabetes. Front. Pharmacol..

[85] Adela R., Nethi S.K., Bagul P.K., Barui A.K., Mattapally S., Kuncha M., Patra C.R., Reddy P.N., Banerjee S.K. (2015). Hyperglycaemia enhances nitric oxide production in diabetes: A study from South Indian patients. PLoS ONE.

[86] Kowluru R.A. (2003). Effect of reinstitution of good glycemic control on retinal oxidative stress and nitrative stress in diabetic rats. Diabetes.

